# Pediatric Hospitalist Resuscitation Skills Refresher Training With Pauses for Deliberate Practice

**DOI:** 10.7759/cureus.20538

**Published:** 2021-12-20

**Authors:** Lana Ismail, Priti Bhansali, Kevin M Creamer

**Affiliations:** 1 Pediatric Hospital Medicine, Children's National Hospital, District of Columbia, USA

**Keywords:** peer learning, pals, pediatric hospitalist, simulation education, pediatric resuscitation

## Abstract

Introduction

Pediatric hospitalists are expected to lead resuscitative efforts for cardiopulmonary arrests, but the infrequency of these events and pediatric advanced life support (PALS) re-certifications are insufficient to maintain skill proficiency.We created a novel resuscitation refresher curriculum for pediatric hospitalists with strategic pauses during simulations for expert and peer coaching of procedural skills.

Methods

In a tertiary care academic pediatric hospital between September 2018 to June 2019, pediatric hospitalists and fellows voluntarily participated in a series of three quarterly two-hour training sessions taught by expert peer facilitators. Sessions focused on the thirty-second rapid cardiopulmonary assessment and each of the pediatric advanced life support (PALS) algorithms. Scenarios were strategically paused to practice critical hands-on skills. Cases centered on the themes of shock, respiratory, and cardiac emergencies and took place in a high-fidelity simulation lab requiring a technician and expert peer facilitator. Participants anonymously completed Likert scale-based evaluations after each session and again at the end of the year that focused on participants’ own perceived change in their comfort levels in performing various resuscitation skills and in knowing basic resuscitation steps. As part of our institutional and personal assessment of the curriculum, an end-of-year survey additionally asked participants to reflect on the overall simulation curriculum and resultant changes in their clinical practice.

Results

Comfort in all skills practiced across the three sessions increased. The end-of-year survey showed a significant rise in comfort above baseline but some decrements when compared to that immediately post-training. Ninety-six percent of pediatric hospitalists rated the overall quality of the training “better” or “much better” than other resuscitation training (including PALS classes and traditional simulations with skills training after the scenario). The overall effect of the curriculum on perceived knowledge, skills, and confidence levels was significant (p <0.0001).

Conclusion

Serial resuscitation skills refreshers with expert peer coaching and strategic pauses for hands-on skills practice can result in significant improvements in perceived knowledge and comfort with skill performance as well as the leadership role among pediatric hospitalists.

## Introduction

Despite ample opportunities for pediatric resuscitation simulation training at academic institutions, pediatric hospitalists are rarely the target participants. Students and residents, however, can usually expect to participate in various forms of resuscitation simulation training regularly. Due to time pressure and a sheer number of participants, these crowded training sessions usually don’t provide opportunities for individuals performing skills to be coached to achieve an ideal standard. The average pediatric hospitalist in a busy practice may only be afforded resuscitation refresher training every two years as part of pediatric advanced life support (PALS) certification. Although the prevalence of pediatric inpatient cardiopulmonary arrests is rare [1], the pediatric hospitalist is still expected to competently take charge of the team and manage the resuscitation for at least the first five minutes until a more robust code team response can be organized [2,3]. This leadership requires proficiency in various individual hands-on skills, knowledge of the PALS resuscitation algorithms, and the ability to direct, observe and correct team performance. Pediatric hospitalists practicing at smaller community hospitals may be expected to lead the resuscitative efforts through a post-resuscitative phase until critical care transport arrives, which can be a daunting proposition when they feel out of practice.

The resuscitation literature has many examples of poor performance during cardiopulmonary resuscitation (CPR) even by “experts” [4,5], the decay of skill after training within six months [6], and how refresher training with mock codes enhances skill retention, knowledge, simulated performance, and comfort level [7-9]. In a recent publication, the American Heart Association highlighted new specific recommendations based on resuscitation education science [10]. They called for deliberate practice and mastery learning for resuscitation training and tasks. They also touted the benefits of booster training and spaced learning in small groups to optimize CPR skill retention and performance over time. This builds on the American Heart Association (AHA)’s 2015 resuscitation quality improvement initiative that was created in recognition that training every two years is suboptimal. This initiative emphasized that more frequent training, including cognitive and psychomotor skills using simulated cases, would be helpful for physicians who may encounter patients in cardiac arrest [11].

Several pediatric resuscitation simulation studies have been published, most of which follow one of two formats: uninterrupted cases with post-scenario debriefing [12-14] or rapid cycle deliberate practice (RCDP), which provides increasingly challenging cases with pauses for directive feedback during and after each resuscitation [15-17]. Similar to some RCDP scenarios, this curriculum embeds deliberate pauses during scenarios for expert-guided skills practice. Unlike RCDP scenarios, this curriculum pauses scenarios before each skill is performed to ensure that correct performance is done from the beginning with expert guidance and peer observation until participants are comfortable with that skill. Rather than allowing participants to perform a skill and then pause to correct their technique as in RCDP, the unique approach we took was based on the philosophy that correct performance of each skill from the start is important to ensure that participants become accustomed to only utilizing the correct technique. This approach was highly regarded shortly after implementation in this setting. Also, instead of primarily targeting the pediatric resident, as the learner as in most studies, this curriculum was created for the attending pediatric hospitalist level provider. It covers all the PALS algorithms, addressing the most likely scenarios a pediatric hospitalist may face in the critical first five minutes of resuscitation.

Our quarterly resuscitation curriculum for pediatric hospitalists and fellows taught in a non-threatening environment to optimize skill performance with deliberate pauses for expert and peer coaching was recently outlined in an opinion piece [18]. This new curriculum with frequent training of hands-on skills aimed to improve pediatric hospitalists’ comfort in performing resuscitations, their knowledge, and psychomotor proficiency.

## Materials and methods

Development

This pediatric hospitalist resuscitation refresher curriculum entailed a series of three quarterly two-hour small-group simulation-based training sessions. The scenarios were designed for use in a high-fidelity simulation lab with a technical support person, simulation facilitator, and no more than four participants per session. Target participants included pediatric hospitalist attending physicians, advanced practice providers, pediatric hospital medicine fellows, and pediatric chief residents. Participants were all from the same hospital medicine division but worked at seven different clinical sites: a tertiary care center and six community sites. All simulations occurred in the tertiary care center’s simulation center.

The simulation facilitator was a pediatric hospitalist with prior critical care experience; however, this role could ideally be filled by any experienced pediatric hospitalist in the division comfortable facilitating for their peers. Having a peer facilitator, as opposed to a specialist from another division, was intentional in order to decrease the social anxiety surrounding an already stressful situation to allow participants to maximally focus on their learning objectives. The facilitator was the primary person who provided the introductory presentation, described the scenario, ran the cases, coached the skills practice, and led the debriefs. During the cases, the facilitator also verbalized exam findings that the mannequin could not demonstrate and interjected with occasional phrases as indicated in each scenario summary.

In addition to the facilitator, a technical support person skilled in managing high-fidelity simulators assisted with each case. Their role was to set up equipment and manage the mannequin’s change in vital signs in real-time.

Study design and equipment

Simulations were run in a high-fidelity sim center at a tertiary care children’s hospital from September 2018 to June 2019. Simulations primarily utilized the Laerdal SimBaby (Maharashtra, India) and the PRESTAN Infant Manikin (Mayfield, Ohio, US) with cardiopulmonary resuscitation (CPR) monitor. Basic resuscitation equipment, including a full code cart, airway supplies, suction, intravenous (IV) fluid supplies, intraosseous (IO) equipment, defibrillator, and emergency medications, was used. Some scenarios also utilized diagrams and de-identified chest X-rays (CXRs) and electrocardiograms (ECGs) as supplements for the skills or to assist in case progression.

Implementation

Participants were incentivized to participate as part of their academic goals linked to their end-of-year bonus, and they signed up voluntarily. During each quarter, a two-hour session was offered a total of 10-14 times, usually twice a day during five to seven days that were randomly distributed over two months. Simulation dates were determined according to the facilitator’s availability, and the same facilitator-led each of these sessions. Participants were allowed to sign up for any date that worked for their schedule, making each group of four participants per session random and different in each quarter. Four was considered the maximum number of participants per session to allow each to actively participate in every simulation case and to practice each skill under the facilitator’s supervision. Some participants only attended a session during one quarter, and others attended a session during two or all three quarters. Each quarter of simulations focused on a different skill set, with quarter one focusing on basic CPR skills, quarter two on respiratory skills, and quarter three on cardiac skills. Participants consisted mostly of pediatric hospitalists with some pediatric hospital medicine fellows and pediatric chief residents. 

Prior to attending their scheduled simulations, participants were encouraged to review the PALS algorithms and other pertinent handouts. At the beginning of each session, the facilitator briefly reviewed CPR pearls and physiology using a 15-minute Microsoft PowerPoint presentation. After a brief orientation describing the goals of the curriculum and mannequin functionality, the scenario stem was read, and the case was initiated.

When the simulation outline indicated a “PAUSE to demonstrate”, for example, after participants stated they’d like to give a push-pull bolus, the entire group stopped the scenario, and the facilitator asked for a participant to demonstrate the skill correctly. Then, each participant performed the skill while being observed and given real-time coaching and feedback by the facilitator and their peers. Special emphasis was placed on the performance of the rapid cardiopulmonary assessment, chest compressions, and bag-valve-mask ventilation (BVM). One participant in each group was given a skill-specific assessment checklist to check themselves and facilitate coaching of peers during their skill performance. This included checklists for the rapid cardiopulmonary assessment, IO placement, and endotracheal intubation, where applicable. These checklists were intended to be cognitive aides to prompt optimal skills performance but not for objective measurements or scoring. After each participant had practiced skill to their and the facilitator’s satisfaction, the scenario was returned to live-action.

Scenarios

**Figure 1:**
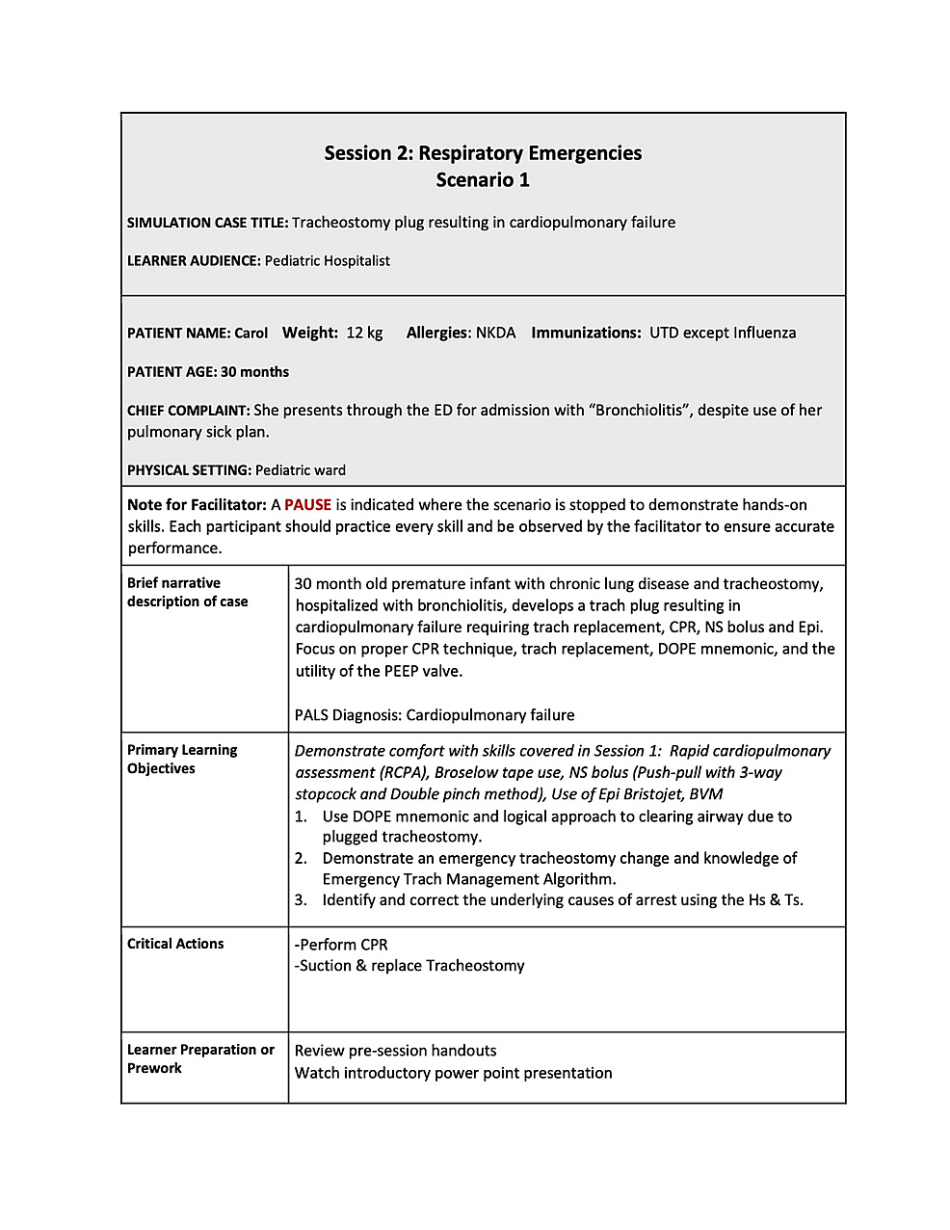
Session 2: Respiratory Emergencies - Scenario 1 page 1

**Figure 2:**
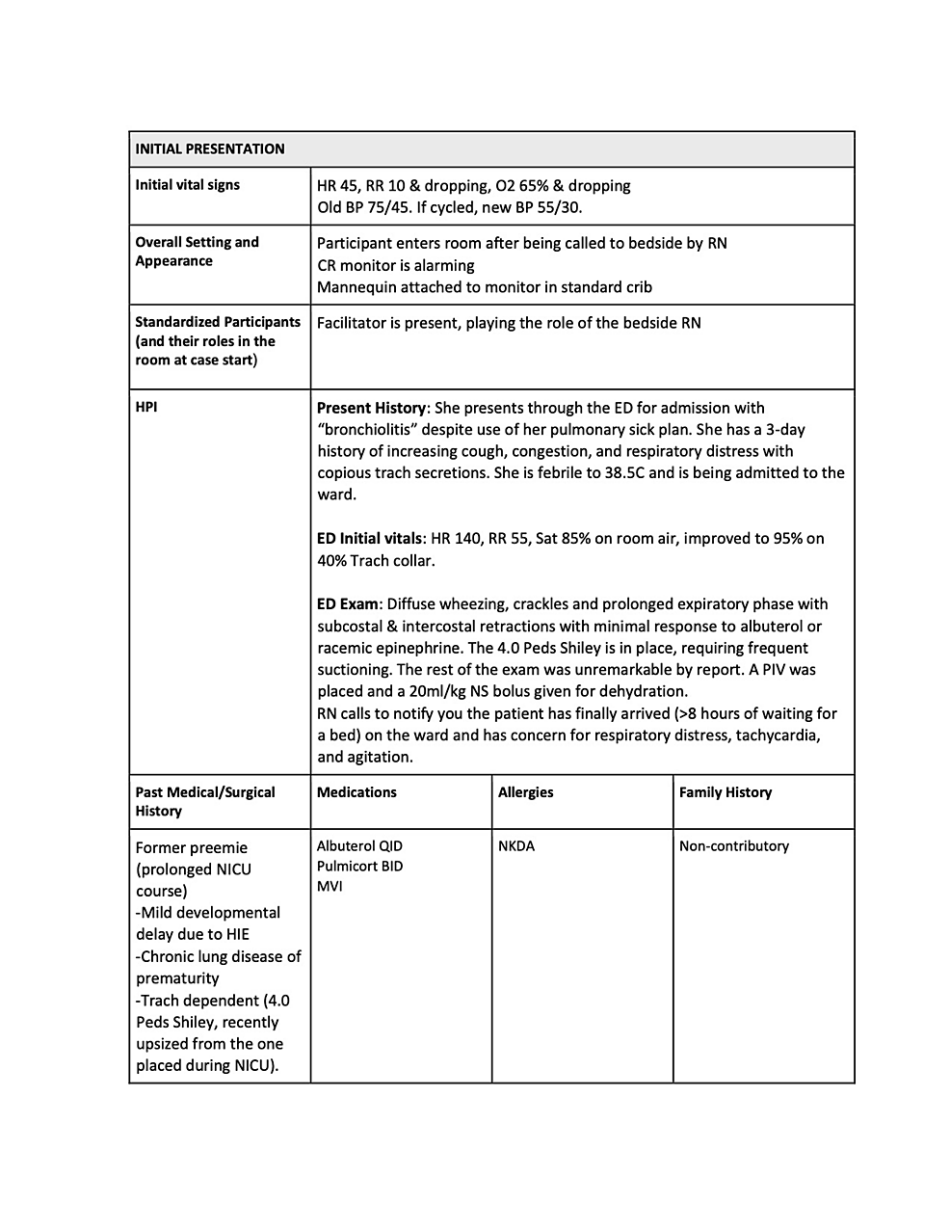
Session 2: Respiratory Emergencies - Scenario 1 page 2

**Figure 3:**
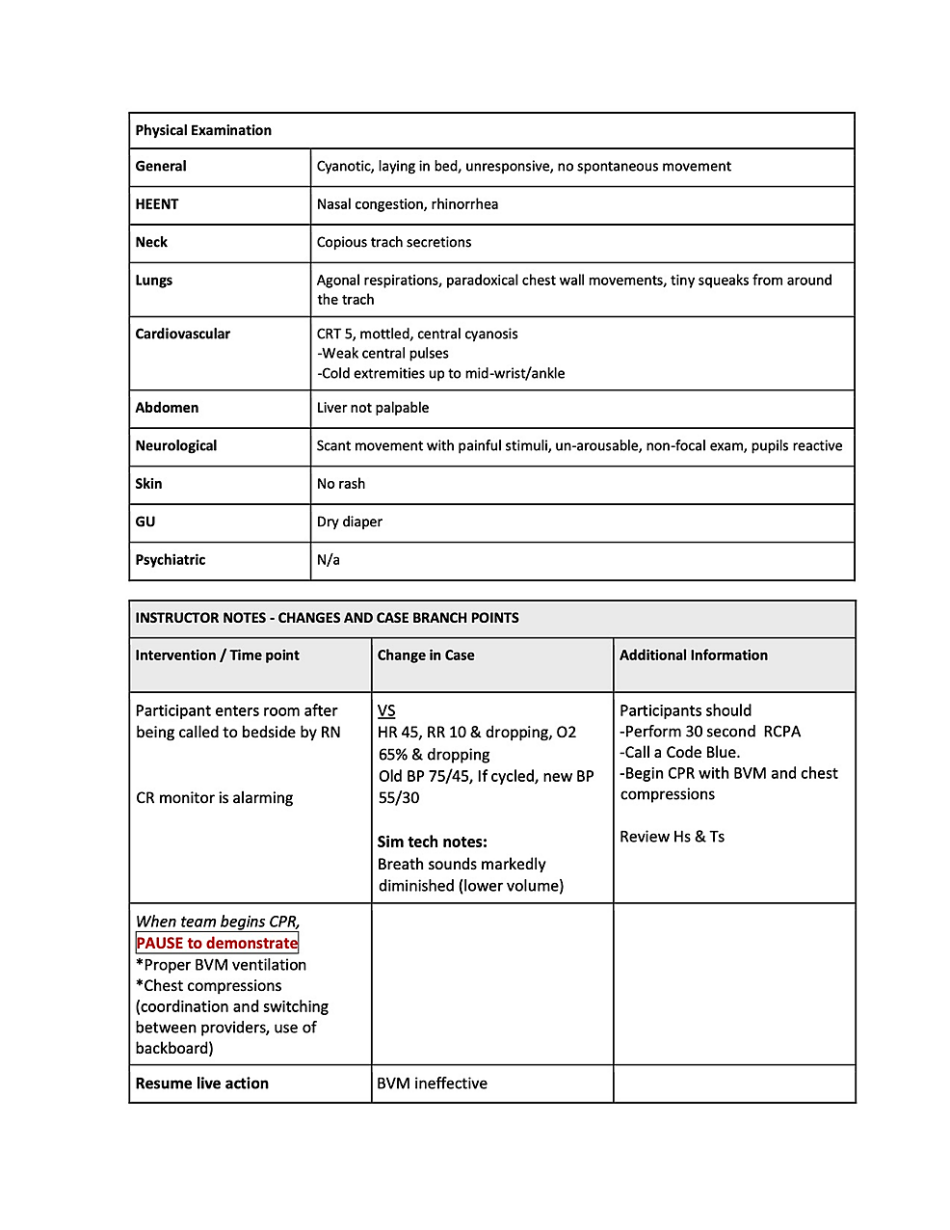
Session 2: Respiratory Emergencies - Scenario 1 page 3

**Figure 4:**
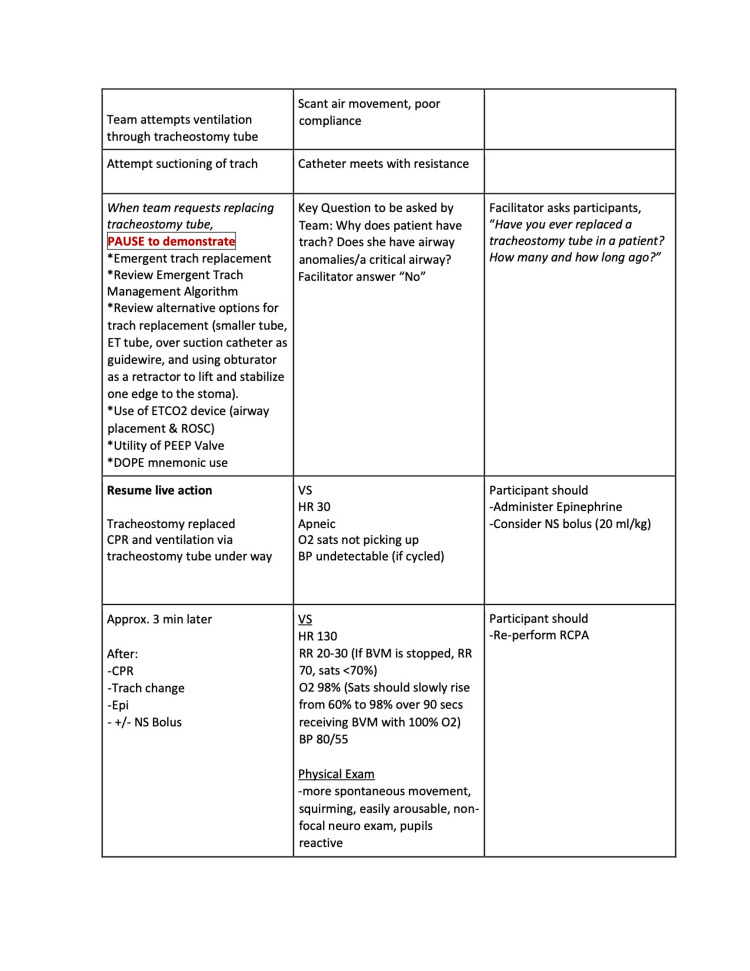
Session 2: Respiratory Emergencies - Scenario 1 page 4

**Figure 5:**
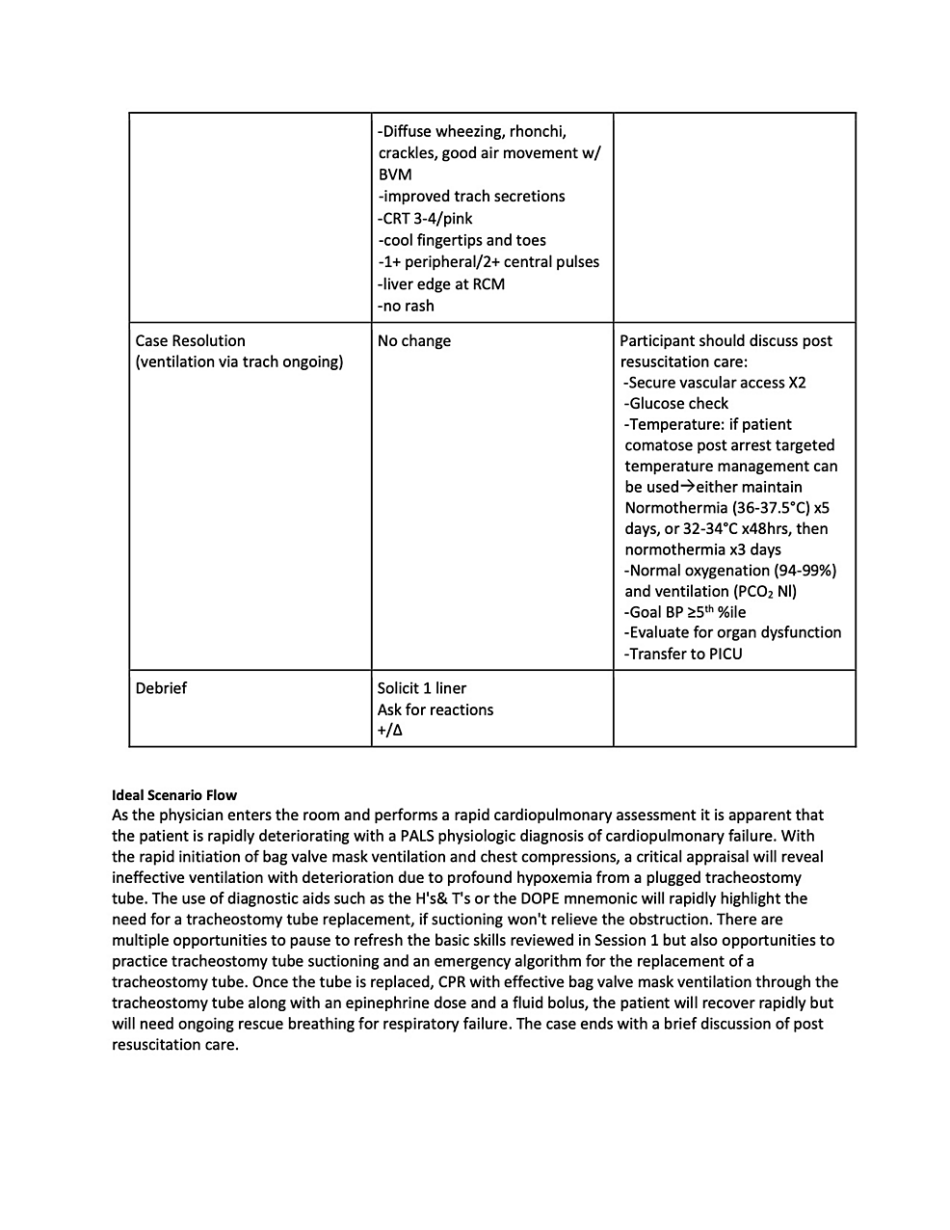
Session 2: Respiratory Emergencies - Scenario 1 page 5

**Figure 6:**
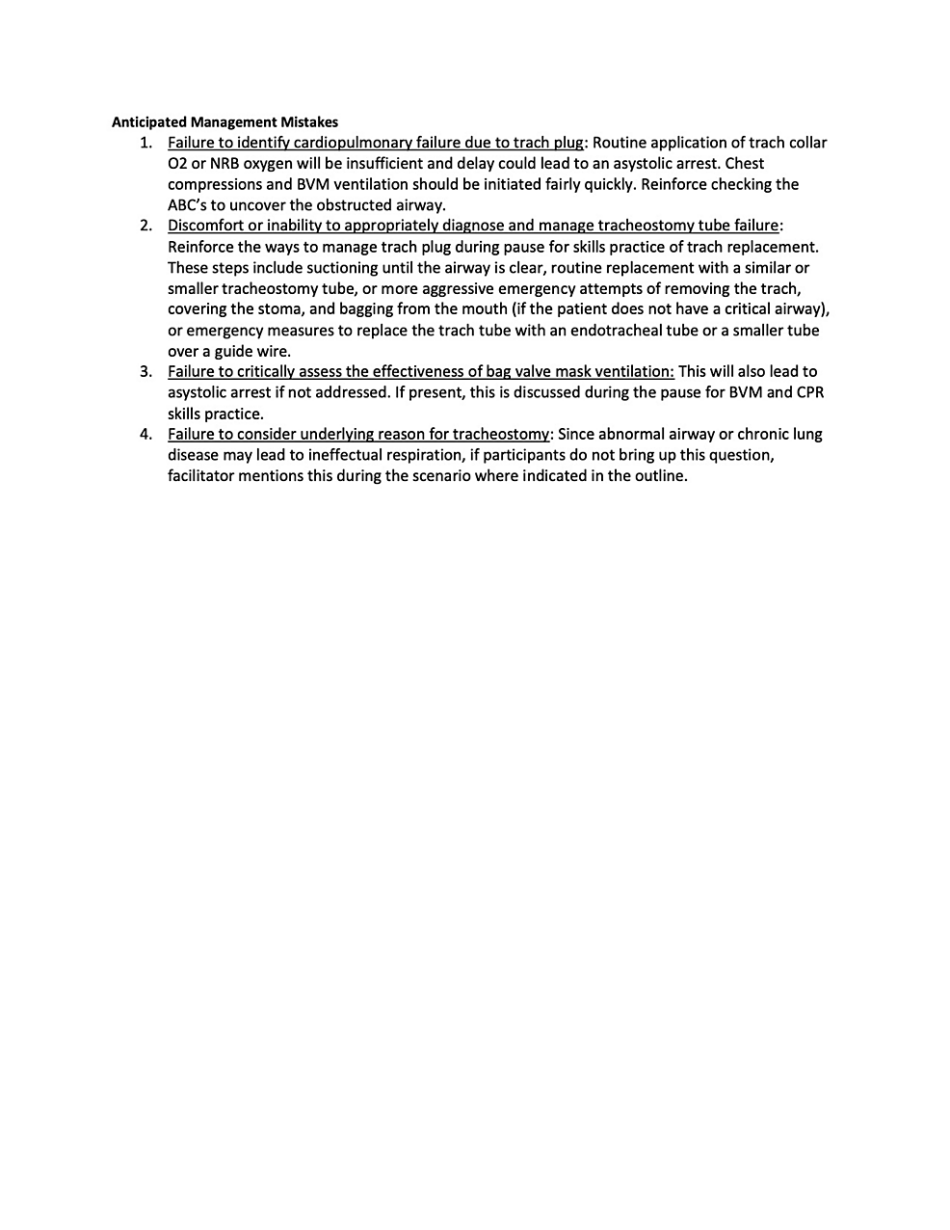
Session 2: Respiratory Emergencies - Scenario 1 page 6

The three quarters within the curriculum focused on cases that highlight basic resuscitation skills and enabled a detailed exploration of the practical hands-on skills involved. Each session had 2-4 scenarios, which built on and reinforced the skills highlighted in prior scenarios. The second quarter’s first scenario is included below (Figures 1-6). 

The first quarterly session focused on hypovolemic shock leading to cardiac arrest requiring CPR. The 30-second rapid cardiopulmonary assessment was reinforced in each scenario of this session and throughout the curriculum. The second session covered the initial management of respiratory failure, emphasizing the importance of appropriate bag-valve-mask ventilation technique, considerations surrounding endotracheal tube intubation, and troubleshooting with emergent replacement of an obstructed tracheostomy tube. The third session highlighted the management of cardiac emergencies due to rhythm disturbances. Each session built on the prior by reinforcing high-quality CPR techniques.

Debriefing

At the end of each scenario, the facilitator led the debriefings, and if there were at least three participants, one was asked to observe before initiation of the case to take notes, evaluate the performance of individual skills using a checklist as mentioned above, and assist with a debriefing of their peers. The facilitator used a Debriefing Guide, which was based on the promoting excellence and reflective learning in simulation (PEARLS) healthcare debriefing tool [19]. After reactions and a one-line case summary were elicited, the debrief was a mix of directive feedback for specific skills, and either a plus/delta (i.e., what went well and what could be changed) or guided advocacy inquiry regarding overall performance during the simulation. The debrief ended with participants vocalizing their personal "take-aways" from the case.

Afterward, participants were given handouts and checklists for their own reference as a “toolkit” of resources to review the skills and concepts covered in the simulation, as well as to enable them to teach those skills to others more effectively.

Statistical analysis

**Figure 7:**
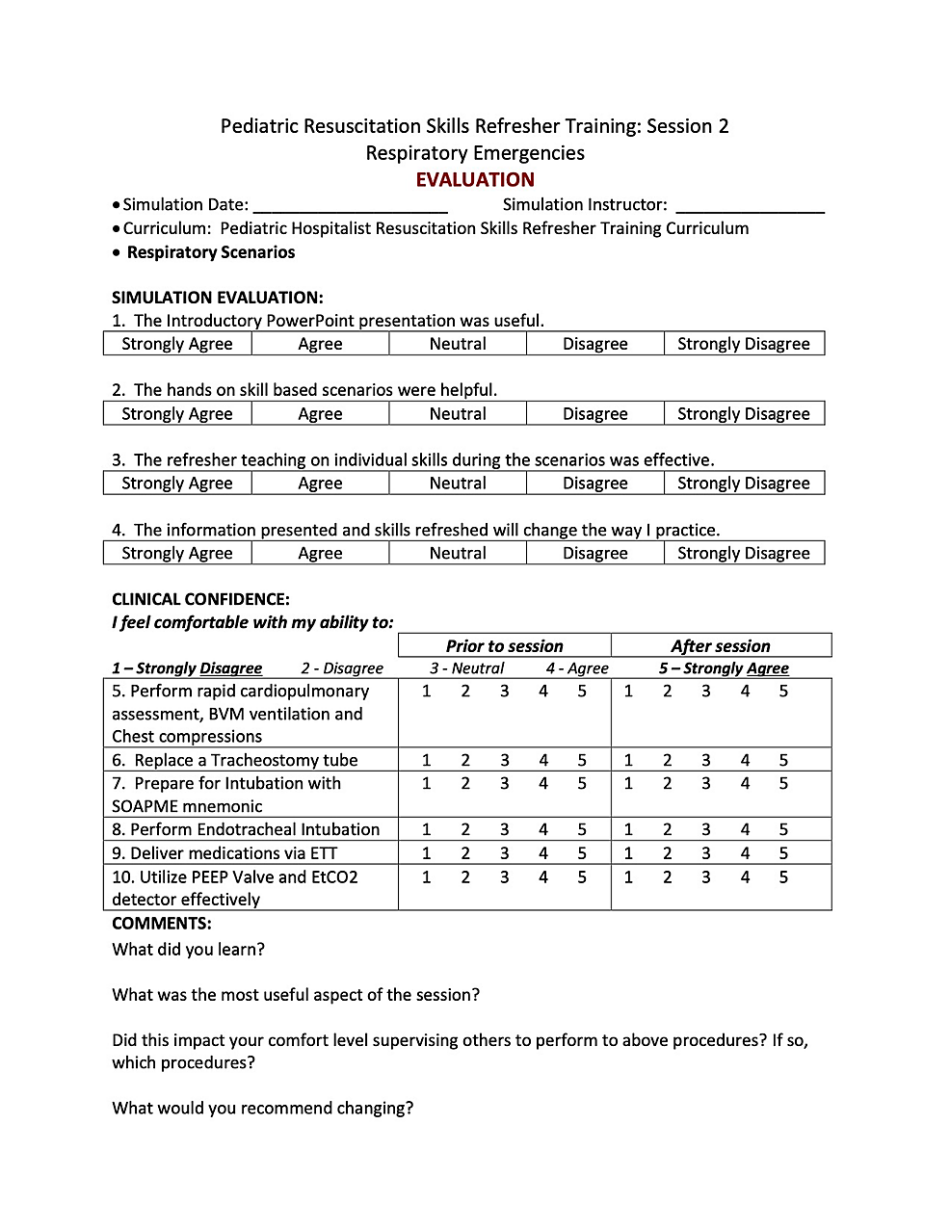
Session 2 Evaluation

Immediately after each session, we distributed an anonymous five-point Likert-scale evaluation to each participant. See the below evaluation for session 2 as an example (Figure 7).

As we were modifying a longstanding survey instrument routinely used for simulations at our institution, we did not collect additional validity or reliability evidence for this tool. Evaluations contained 9-10 Likert-scale questions and four free text questions. Several of the Likert-scale questions asked participants to rate their perceived comfort levels with performing specific resuscitation skills before the session and compare it with their current comfort level immediately after the session. These questions changed every quarter to highlight the different skills that were practiced during each quarter’s simulations. Other questions asked participants to rate their confidence in leading a code and to discuss whether they had gained comfort in teaching any of the skills they had learned.

**Figure 8:**
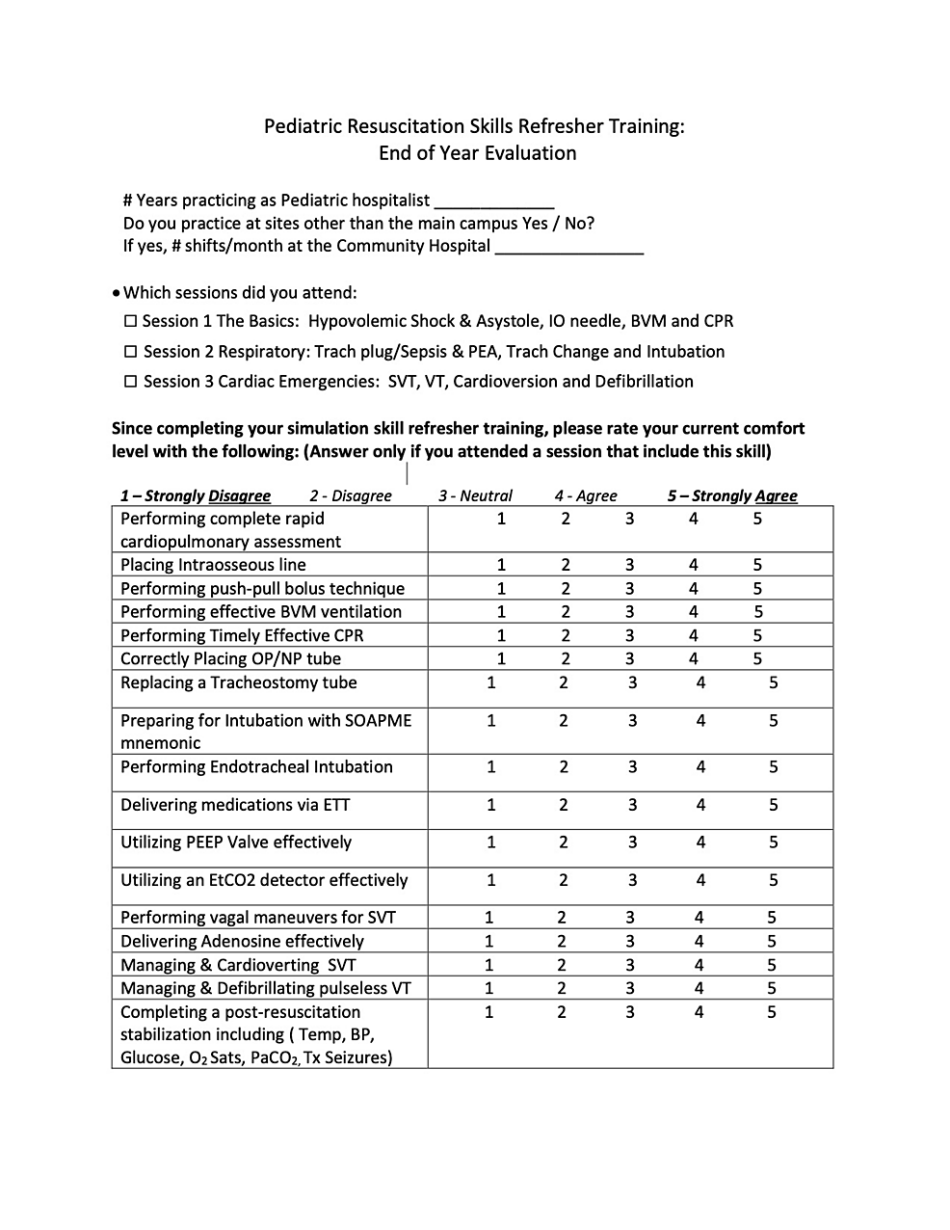
End of Year Evaluation page 1

**Figure 9:**
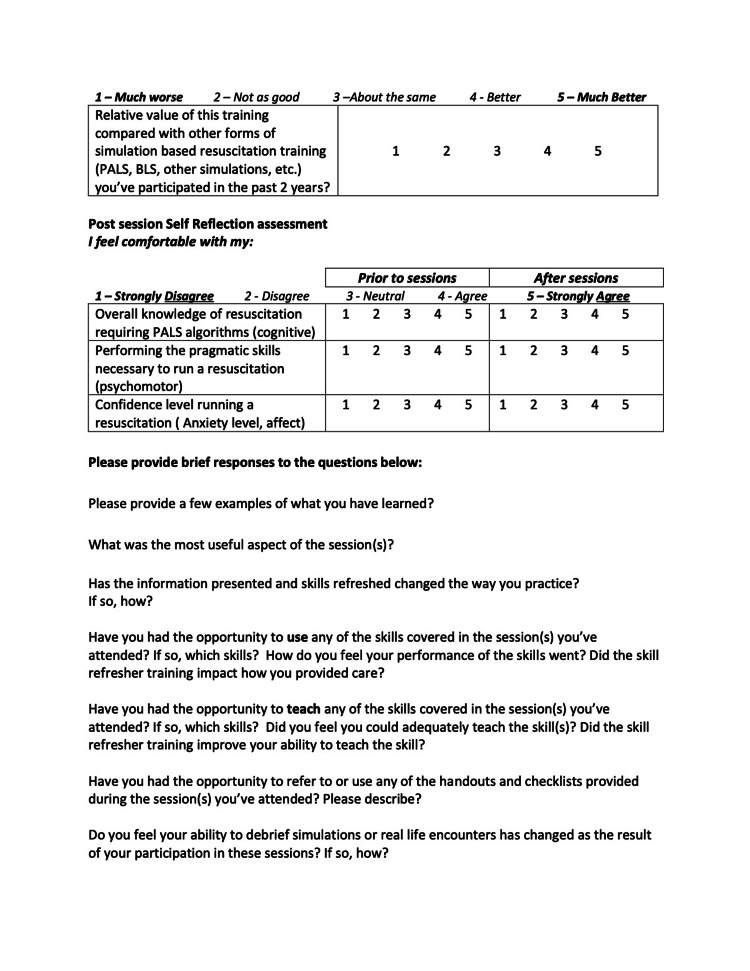
End of Year Evaluation page 2

A few months after the three quarterly sessions were completed, an anonymous “End of Year Evaluation” was also distributed to participants who attended any or all sessions (Figures 8, 9).

This survey was part of our personal and institutional simulation program’s assessment of the curriculum to evaluate the curriculum’s overall effect beyond the immediate post-session reflections and identify ways to improve the value of the simulation program. This survey contained 21 Likert-scale questions that asked about participants’ current comfort levels in performing all of the skills covered in the three sessions of the curriculum. It also contained seven free text questions that probed whether participants had made changes in their practice after participating in this simulation curriculum and asked for further reflections on the curriculum.

Comfort levels with performing each procedure or resuscitation skill were scored on a scale of one to five; where one indicated the participant “strongly disagreed”, and five indicated the participant “strongly agreed” with feeling comfortable performing the procedure. For each session, the comfort level with each procedure was compared before and after the session using a paired t-test. The mean of the responses from each training session was analyzed using a one-way analysis of variance (ANOVA). Pre-training comfort levels with performing the complete rapid cardiopulmonary assessment across three sessions were also analyzed using ANOVA. Comfort levels with the rapid cardiopulmonary assessment across all three sessions were analyzed using the analysis of covariance (ANCOVA), treating the pre-training comfort level as a covariate and post-training comfort level as the response.

While the infrequency of pediatric resuscitation events made actual patient-level objective outcomes difficult to obtain, we solicited testimonials about how this simulation curriculum may have affected participants’ management in resuscitation events. These testimonials were shared by participants both in the end-of-year survey as well as ad hoc as the events occurred.

## Results

Among the 89 pediatric hospitalists invited to these sessions, an average of 35 participants attended each of the three quarterly sessions (44, 29, and 32 participants in the first, second, and third sessions, respectively).

After the first quarter’s sessions, respondents rated their comfort levels in placing an IO line, performing push-pull boluses, effective BVM ventilation, timely effective CPR, and correctly placing an OP/NP tube compared to before attending the session. The average increase between pre- and post- comfort levels on a five-point Likert scale was 1.4 (p <0.0001). Similarly, after the second quarter’s sessions, comfort levels in replacing tracheostomy tubes, preparing for and performing intubation, and delivering medications via endotracheal tube (ETT) also increased (mean 1.2, p <0.0001). After the third quarter’s sessions, comfort in performing vagal maneuvers for supraventricular tachycardia (SVT), delivering adenosine, managing and cardioverting SVT, managing and defibrillating pulseless ventricular tachycardia (VT), and completing a post-resuscitation stabilization also increased an average of 1.3 points (p <0.0001). Similar trends were seen when participants rated their comfort levels at the end-of-year survey. However, overall improvement in comfort levels at the end of the year compared to immediately post-session was slightly lower (Table 1).

**Table 1:** Evaluation results prior to and after Sessions 1-3 and End of year survey comparison Comparison of comfort levels performing specific skills based on 5-point Likert scale ranging from “strongly agree" = 5 to “strongly disagree” = 1

I feel comfortable with my ability to:	Sessions 1-3 surveys	Prior to training	Immediately after training	Comparing prior and after		End of year survey	End of year vs prior to first session	
(Strongly Agree = 5, Strongly Disagree = 1)	N	Mean	Mean	Mean Difference	p-value	N	Mean Difference	p-value
Session 1: The basics								
Place Intraosseous line	44	2.82	4.39	1.57	<0.0001	27	1.1448	< .0001
Perform push- pull bolus technique	43	2.44	4.47	2.02	<0.0001	27	1.5211	< .0001
Perform effective BVM ventilation	44	4.02	4.81	0.80	<0.0001	26	0.6696	0.0002
Perform timely effective CPR	43	3.47	4.60	1.14	<0.0001	26	1.1381	< .0001
Correctly place OP/NP tube	44	2.86	4.41	1.50	<0.0001	25	0.8964	0.0008
Session 2: Respiratory emergencies								
Replace a tracheostomy tube	29	3.24	4.52	1.28	<0.0001	25	0.8386	0.0092
Prepare for intubation with SOAP-ME mnemonic	29	3.03	4.38	1.34	<0.0001	24	0.8822	0.0004
Perform endotracheal intubation	29	3.24	4.21	0.97	<0.0001	24	0.6336	0.0123
Deliver medications via ETT	27	2.81	3.93	1.11	<0.0001	24	0.7619	0.0022
Session 3: Cardiac emergencies								
Perform vagal maneuvers for SVT	32	3.38	4.69	1.31	<0.0001	27	1.0324	< .0001
Deliver Adenosine effectively	32	3.59	4.66	1.06	<0.0001	26	0.7524	0.0013
Manage & Cardiovert SVT	32	3.13	4.56	1.44	<0.0001	26	1.0288	< .0001
Manage & Defibrillate pulse less VT	32	2.69	4.19	1.50	<0.0001	26	1.3125	< .0001
Complete a post resuscitation stabilization	32	3.34	4.50	1.16	<0.0001	25	0.8963	< .0001

Among the 29 participants responding to the end-of-year survey, the vast majority had attended all three quarterly sessions. They rated an overall 1.2-point increase in their comfort in performing the rapid cardiopulmonary assessment (Table 2).

**Table 2:** Evaluation of comfort with rapid cardiopulmonary assessment prior to and after Sessions 1-3 and in the end-of-year survey Comparison of comfort levels based on a 5-point Likert scale ranging from “strongly agree" = 5 to “strongly disagree” = 1

I feel comfortable with my ability to:	Session Survey (N)	Prior to	Post	Comparing Prior and Post	Pre-training across three sessions	End of year vs before Session 1
(Strongly Agree = 5, Strongly Disagree = 1)		Mean	Mean	p-value	p-value (ANOVA)	
Perform a complete rapid cardiopulmonary assessment	1 (44)	3.34	4.52	<0.0001	0.0081	n/a
	2 (29)	3.83	4.69	<0.0001		
	3 (32)	3.78	4.75	<0.0001		
	End of year (27)	4.56	n/a	n/a	n/a	<0.0001

While most participants had not had the opportunity to use the skills refreshed in these sessions on an actual patient, a few reported teaching these skills with greater comfort. Overall comfort in performing the pragmatic resuscitation skills and confidence in running a resuscitation each increased by 1.4 points (p <0.0001) (Table 3).

**Table 3:** End-of-year survey: Reflections on the impact of the curriculum

End of year survey		Prior	Post	Comparing Post to Prior	
I feel comfortable with my: (Strongly Agree = 5, Strongly Disagree = 1)	N	Mean	Mean	Mean Difference	p-value
Overall knowledge of resuscitation requiring PALS algorithms (cognitive)	28	3.46	4.50	1.0357	< .0001
Performing the pragmatic skills necessary to run a resuscitation (psychomotor)	28	2.96	4.36	1.3929	< .0001
Confidence level running a resuscitation (anxiety level, affect)	28	2.68	4.04	1.3571	< .0001

Compared to prior simulation training without expert peer coaching or deliberate pauses during scenarios for skills practice, this training was rated “better” or “much better” by 96% of participants. When asked what the most useful aspect of the curriculum was, a typical response included the “hands-on, frequent breaks to debrief/explain/clarify” and the “non-threatening environment among division peers”. Others reported that the “increased confidence in handling code situations is invaluable”. After a recent code event, a colleague recalled, “the repetition of good CPR technique and the H’s/T’s was really helpful in the code…there were so many instances during the code when there was a callback to a simulation lesson that I felt comfortable during the actual management once the code started.” Another colleague assisting in the management of a child in cardiac arrest in a community hospital emergency room stated, “I am grateful that I had knowledge and tools from the simulation to use that allowed me to manage the situation with less fear and uncertainty.” Regarding the most useful aspect of the session, one respondent mentioned “the fact that they build upon each other. I was able to practice skills from prior sessions in the later sessions, which helped solidify my knowledge and skills. These sessions have been fantastic.”

## Discussion

Although the pediatric hospital medicine division had an existing simulation curriculum before this model, facilitators were usually content experts who were not hospitalists, there was no continuity of facilitators, and sessions did not build on prior skills. Hence, hospitalist comfort with resuscitation skills and executing PALS basics was still lacking. Out of concern that this lack of proficiency may affect pediatric hospitalists’ confidence and performance, especially when working at remote community sites, this novel curriculum was tailored to focus on the first five minutes of resuscitation and lifesaving hands-on skills. It served our pediatric hospitalists who practice in various clinical settings across seven different sites.

Key differences between this simulation curriculum and others at the institution and in the literature include training by and for pediatric hospitalists, and the use of guided deliberate pauses for skills practice during scenarios utilizing expert peer facilitators. Having pauses occur before skills are performed in a scenario reinforces the correct method from the start until a satisfactory performance is achieved according to the peer expert and objectively using skills checklists. This model also resonates with the AHA's new resuscitation science education emphasis on deliberate practice and mastery learning with booster training spaced over time [10]. Our results reflect the benefit of this approach given the serial and persistent documented improvements in the RCPA over time. While there were slight decrements in comfort levels with resuscitation skills at the end of the year compared to immediately after each session, the sustained overall comfort with resuscitation skills may be attributed to repeated practice in cases that built on prior skills and emphasized correct performance.

This curriculum has several limitations. First, it assumes a pediatric hospital medicine division member with resuscitation experience may be willing to lead these sessions. Some pediatric hospitalists may find it challenging to implement this curriculum at their sites if they have no such division expertise, and this curriculum did not address training the trainer. Further, the lack of a high-fidelity simulation lab with a technician able to run the simulations may not take away from the hands-on skills practice, but it may interfere in the suspension of disbelief during these sessions. The two-hour time commitment required for participation may also not be feasible in some practice settings. Support from division leadership by providing paid non-clinical time for the facilitator to run these sessions and linking participation with incentives was instrumental for this project and could be the key to successful implementation at other sites.

Other limitations include the fact that the objectives and evaluations in this study center on comfort levels as opposed to objective measures due to the logistical challenge of collecting objective data. Outcome changes are especially challenging to track as our hospitalists practice at seven different clinical sites, which is why we solicited testimonials at the end-of-year survey about how the curriculum changed their practice. However, only a few participants reported practice change, likely due to the rarity of resuscitation events in pediatric hospital medicine. Although this survey mostly assessed level 1 outcomes in the New World Kirkpatrick Model [20], respondents noting a subjective change in their knowledge and ability to teach the skills they learned indicate some achievement of level 2 outcomes in this model. The next step to gauge the efficacy of this curriculum would be to objectively assess improvement in participants’ resuscitation and skills performance using a validated checklist and evaluating the time to perform each intervention.

Given the overwhelmingly positive reviews after this first year’s curriculum, new curriculum content was developed, now in its third year. Due to the COVID-19 pandemic, the third year of simulations has been all virtual, still emphasizing the use of rapid cardiopulmonary assessment and the Hs and Ts from PALS.

## Conclusions

Although simulation training sessions in this pediatric hospital medicine division existed before our novel curriculum, restructuring sessions by incorporating deliberate pauses during scenarios for practicing skills and reinforcing the basic skills every quarter basis had a more profound effect on pediatric hospitalist resuscitation comfort in performing these life-saving interventions than expected. Other pediatric hospital medicine groups may benefit from this curriculum for providers in the community and tertiary care hospitals alike. This curriculum showed that a recurring, deliberate, and structured approach to simulation and skills practicing can be the key to reducing performance anxiety and increasing pediatric hospitalist confidence in their resuscitation knowledge and psychomotor skills. Our next steps include using a validated checklist to evaluate performance and time to intervention. Additionally, we aim to have videotaped simulation scenarios to allow for more comprehensive debriefing and improve perceived self-efficacy. Future directions also include having on-site community hospital-based team resuscitations that shift the primary focus from performing tasks correctly to integrated teamwork.

## References

[1] Knudson JD, Neish SR, Cabrera AG (2012). Prevalence and outcomes of pediatric in-hospital cardiopulmonary resuscitation in the United States: an analysis of the kids' inpatient database*. Crit Care Med.

[2] American Heart Association (2006). 2005 American Heart Association (AHA) guidelines for cardiopulmonary resuscitation (CPR) and emergency cardiovascular care (ECC) of pediatric and neonatal patients: pediatric basic life support. Pediatrics.

[3] Hunt EA, Walker AR, Shaffner DH, Miller MR, Pronovost PJ (2008). Simulation of in-hospital pediatric medical emergencies and cardiopulmonary arrests: highlighting the importance of the first 5 minutes. Pediatrics.

[4] Abella BS, Alvarado JP, Myklebust H (2005). Quality of cardiopulmonary resuscitation during in-hospital cardiac arrest. JAMA.

[5] Sutton RM, Niles D, Nysaether J (2009). Quantitative analysis of CPR quality during in-hospital resuscitation of older children and adolescents. Pediatrics.

[6] Braun L, Sawyer T, Smith K (2015). Retention of pediatric resuscitation performance after a simulation-based mastery learning session: a multicenter randomized trial. Pediatr Crit Care Med.

[7] Settgast A, Nguyen JT, Devries A, Krebs E, Duane P (2006). An innovative approach to teaching resuscitation skills. Med Teach.

[8] van Schaik SM, Plant J, Diane S, Tsang L, O'Sullivan P (2011). Interprofessional team training in pediatric resuscitation: a low-cost, in situ simulation program that enhances self-efficacy among participants. Clin Pediatr (Phila).

[9] Nadel FM, Lavelle JM, Fein JA, Giardino AP, Decker JM, Durbin DR (2000). Teaching resuscitation to pediatric residents: the effects of an intervention. Arch Pediatr Adolesc Med.

[10] Cheng A, Magid DJ, Auerbach M (2020). Part 6: resuscitation education science: 2020 American heart association guidelines for cardiopulmonary resuscitation and emergency cardiovascular care. Circulation.

[11] (2021). American Heart Association CPR & First Aid Emergency Cardiovascular Care: Resuscitation quality improvement program (RQI). https://cpr.heart.org/en/cpr-courses-and-kits/rqi.

[12] Rideout M, Raszka W (2018). Hypovolemic shock in a child: a pediatric simulation case. MedEdPO.

[13] Reid J, Stone K (2013). Pediatric emergency medicine simulation curriculum: septic shock. MedEdPO.

[14] Bergman CM, Howell J (2020). Critical cardiopulmonary event series: four simulations for pediatric ICU fellows, critical care nurses, and pediatric residents. MedEdPO.

[15] Doughty C, Welch-Horan T, Hsu D (2015). Rapid cycle deliberate practice pediatric simulation scenarios. MedEdPO.

[16] Gross IT, Abrahan DG, Kumar A, Noether J, Shilkofski NA, Pell P, Bahar-Posey L (2019). Rapid cycle deliberate practice (RCDP) as a method to improve airway management skills—a randomized controlled simulation study. Cureus.

[17] Lemke DS (2020). Rapid cycle deliberate practice for pediatric intern resuscitation skills. MedEdPO.

[18] Creamer KM, Ismail L, Smith K (2020). Practice makes better: making the case for a novel hospitalist resuscitation curriculum. Hosp Pediatr.

[19] Eppich W, Cheng A (2015). Promoting excellence and reflective learning in simulation (PEARLS): development and rationale for a blended approach to health care simulation debriefing. Simul Healthc.

[20] Kirkpatrick JD, Kirkpatrick WK (2016). Kirkpatrick’s Four Levels of Training Evaluation. IJMESS.

